# The biogas upgrading from landfill leachate pretreated with low-frequency ultrasonic: anaerobic digestion performances and energy balance

**DOI:** 10.1038/s41598-023-42996-0

**Published:** 2024-01-05

**Authors:** Ali Hosseinzadeh, Saeid Gitipour, Nasser Mehrdadi

**Affiliations:** 1 Department of Environmental Engineering, University of Tehran, Tehran, Iran; 2 Faculty of Environment, School of Engineering, University of Tehran, Tehran, Iran

**Keywords:** Biological models, Biological techniques, Biotechnology

## Abstract

The efficient biogas production from landfill leachate (LL) is one of hot topics in anaerobic digestion systems. Higher bioavailability of LL can be achieved by application a feasible and promising pretreatment technologies in order to utilize as a substrate for anaerobic reactors. Here, the enhanced bioavailabity of LL using the low-frequency ultrasonic process and energy balance in anaerobic digestion process was estimated within incubation period of 24 days. The optimal performance of low-frequency ultrasonic for LL biodegradability index: sCOD and TVFA were estimated under influencing parameters: ultrasonic density (UD) (0.02–0.14 W/mL) and Ultrasonic time (UT) (0–12 min). Moreover, the effects of low-frequency ultrasonic pretreatment process on biogas production in batch mode anaerobic reactors operated at 37 ± 1 °C were surveyed for daily and cumulative methane production, operational performance and energy balance. An increased sCOD (820 mg/L) and TVFA (659 mg/L) were observed under optimum codition: UD (0.1 W/mL) and UT (10 min). The highest methane production (430 mL) was found in reactor 4, where %15 volume ratio of LL pretreated with low frequency ultrasonic were feed in. Energy balance assessment indicated that output energy for anaerobic reactors assissted with ultrasonic in range of + 6.99 and + 7.98 kJ/g VS removed. Therefore, incorporation the low-frequency ultrasonic and digestion process revealed a promising and economic technique to improve biomethane potential and energy balance from LL.

## Introduction

The ever-increasing solid waste generation due to vast urbanization and rapid population growth, especially, in developing countries is known as a governmental challenge and environmental and economical issue^1,2^. Landfilling is the predominant approach for handling the waste management in both developed and developing nations, due to simple and easy operation and energy recovery. However, leachate production with high pollution and toxic substances occurred due to chemical and biological reactions in landfill sites is the major issue, causing human and ecological risk^3,4^. The LL with extremely variable characteristics contains high COD, BOD, ammonia–nitrogen content, recalcitrant inorganics, liable organics and trace of heavy metals^5,6^. As the landfill age increases, the BOD/COD as the biodegradable index of LL experienced a decreasing trend and LL are difficult to deal with conventional treatment process. The biodegradability index for LL with age lower than 5 years are > 0.5, as time proceed, the mature LL has BOD/COD lower than 0.1^7^. As a result of strict legislations, a variety of physical, chemical and biological methods have been developed to treat the landfill before discharge into environment^8^. According to open literature, most physical and chemical post-treatment methods have drawn much attention in order to lower the LL pollution before discharge into environment^4,9, 10^.

For instance, Saurabh M. Josh and et al. (2018) investigated the combined ultrasonic, H_2_O_2_ and Fenton process to treat the landfill leachate. The authors reported that under optimal conditions (H_2_O_2_: 5 g/L and Fe^2^^+^/H_2_O_2_ ratio of 1:10), the COD was reduced by 92%^10^. V. Gowri Maheswari and et al. (2022) reported that combined ozonationa and Fenton process improve the COD removal efficiency of LL by 83% after 75 min reaction time^4^.

As early mentioned, LL with liable organic matters could be considered as source of energy through anaerobic digestion process assisted with pretreatment processes^11,12^. To date, due to limited production of biogas and energy production from landfill leachate, the anaerobic digestion processes have not been welcomed by the researchers and authorities. Furthermore, the presence of inherent inhibitors and lower quantity of microorganisms, which are incapable of breaking down resistant substances, are additional factors contributing to the limited use of anaerobic digestion in the treatment of LL^13–15^. However, pretreatment the LL prior the feeding into anaerobic digestion can intensify the hydrolysis as the rate-limiting stage in digestion process. The pretreatment process can promote the recalcitrant compound degradation and increase the bioavailability of substrate^16,17^. Although a variety of different pretreatment methods including physical, chemical, mechanical and biological techniques (or combination of these) have been developed to upgrade the biomethane potential (BMP) from different substrates such as organic waste and sewage sludge^18,19^, the pretreatment of LL as the sole substrate has not grasped much attention due to lack of sufficient capable microorganisms to degrade the volatile fatty acids.

The low-frequency sonication is known as a relatively new technology for pretreatment of substrates before feeding the biological reactors^3,20^. The low-frequency sonication with advantages of high decomposition speed, no secondary pollution, easy operation and simple equipment is suggested as alternative to other complex pretreatment process for sewage sludge, industrial wastewater and LL in order to enhance the biological treatment process^1,21^. Generally, sonication process is attributed to cavitation phenomenon which occurs in four consequential stages including nucleation, growth, violent collapse of cavitation bubbles and transient of vapor bubbles to the liquid medium^9^. As a results of collapse of cavitation bubbles, occurrence of extreme conditions including temperature higher than 5000 K and pressure more than 100 MPa contributed to many physicochemical effects such as highly reactive OH^-^ radicals generation^22,23^. Hydroxyl radicals react with recalcitrant organic compounds found in the LL and increase the bioavailability of compounds which are suitable for further anaerobic digestion and biogas production^8,24^. Furthermore, the introduction of anaerobic digested sludge into the reactors harnesses microorganisms with the ability to decompose stubborn substances and are resilient to various chemical and thermal stresses, has been demonstrated to enhance biogas generation during the anaerobic digestion procedure^25,26^. As a result, the current research was conducted to assess the increase in biogas production from LL with the assistance of low-frequency ultrasonic technology. The some other aims of current research were: (1) Evaluate the functional efficiency of the anaerobic digestion procedure during the incubation phase., and (2) determination the energy balance of anaerobic digestion in terms of energy production and energy consumptions.

## Methods and materials

### Material characteristics

For this particular investigation, the LL samples were collected from the Aradkooh municipal solid waste facilities (AMSWF), receiving 5500 tons per day of waste. The LL samples were filtered to eliminate any large particles and then promptly transported to the laboratory where they were stored at 4°C until subsequent analysis. Table 1 summarizes the physico-chemical properties of raw LL used in the present study. The digested sludge as an inoculum samples were also prepared from the full-scale anaerobic digestion operated at mesophilic condition and 37 ± 1 °C located in south Tehran wastewater treatment plant (STWWTP). It is important to note that, the inoculum were initially incubated at mesophilic condition in order to reduce the background biogas production. The physico-chemical characteristics of inoculum are summarized in Table 1.

**Table 1:** Physico-chemical characteristics of raw landfill leachate and inoculum used for anaerobic digestion process.

Parameter	Unit	Inoculum	Leachate
pH	–	7.68 ± 0.2	7.1 ± 0.2
DS	%	2.86 ± 0.1	1.7 ± 0.01
VS/DS	%	71 ± 2	85 ± 1
sCOD	mg/L	1556 ± 25	13,560 ± 50
TVFA	mg/L	1130 ± 10	10,530 ± 25
Alkalinity	mg/L	7950 ± 22	52,854 ± 91

### Ultrasonic pretreatment experiments

**Figure 1:**
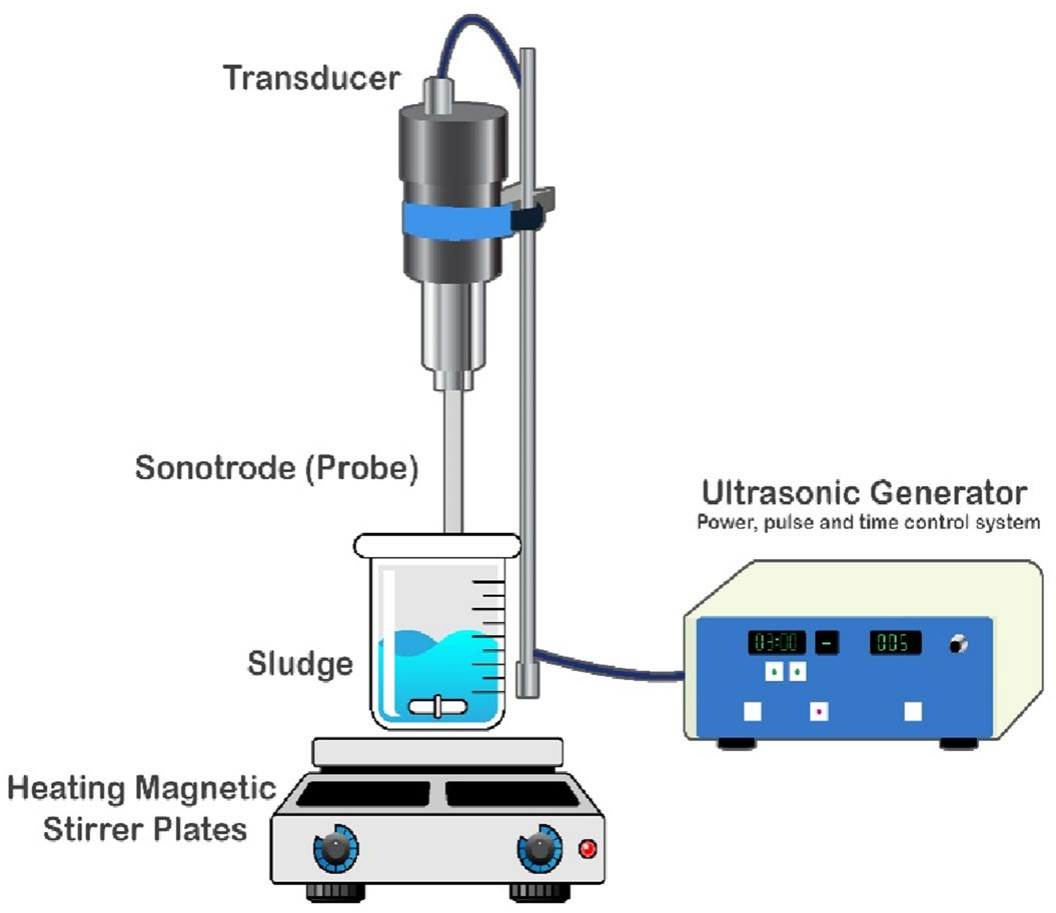
The diagram illustrating the ultrasonic pre-treatment process.

The experimental low-frequency ultrasonic runs were performed to investigate the bioavailability of LL (sCOD and TVFA) before feeding to anaerobic reactor. To this end, the experimental studies using a cylindrical reactor outfitted with a 3 cm diameter probe (Bandelin-SonopulsSonopuls GM model 2070, Germany) were conducted at a low frequency of 20 kHz and a power output of 70 W. Furthermore, the impact of ultrasonic density (UD) (W/mL) and irradiation time (UT) (min) on improving the bioavailability of LL was explored. 500 mL raw landfill leachate were poured in beaker and the effects of ultrasonic densities (UD) (0.02, 0.04, 0.06, 0.08, 01, and 0.12 W/ mL sample) on LL bioavailability were surveyed. A schematic of ultrasonic reactor is presented in Fig. 1.

### Anaerobic digestion process

**Figure 2:**
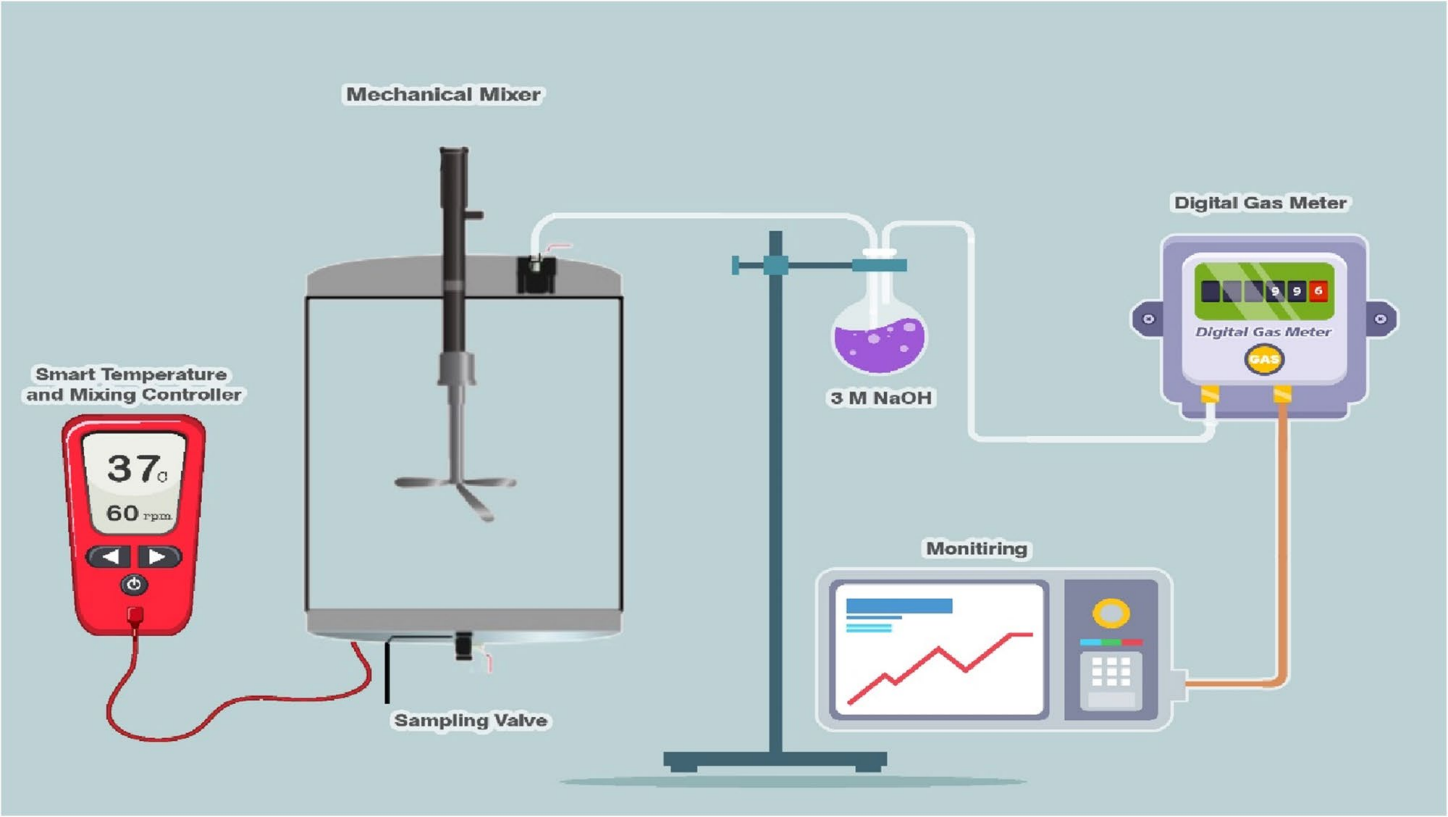
A schematic of anaerobic reactors feed with landfill leachate pretreated with low-frequency ultrasonic.

The biomethane potential (BMP) corresponded to LL pretreated by low-frequency ultrasonic pretreatment were investigated via the completely mixed anaerobic batch reactors with incubation period of 24 days. To this end, a total of 4 plexiglass reactors, each containing 2 and 1 L total and working volume, respectively were fed with different volume ratio of LL (10 and 15%) pretreated with low-frequency ultrasonic and controls and operated at mesophilic temperature (37 ± 1 °C). A hot water circulation equipped with sensitive sensor was used to maintain the mesophilic temperature during the incubation period. To make assurance of anaerobic situation, the pure nitrogen gas were purged into reactors for 5 min at the start of incubation period. A mechanical mixer, operating in a two-stage configuration with 5 min on and 1 min off, was employed to facilitate mixing within the reactor at a speed of 60 rpm. The methane gas volume was measured using a digital gas meter, calibrated beforehand using the water displacement technique. The H_2_S and CO_2_ as the other components of biogas produced from each anaerobic reactors were removed by movement the biogas from a container with alkaline solution (3 M NaOH). The diagram presented in Fig. 2 illustrates the schematic layout of the anaerobic reactors that were utilized in this study. In addition, the other operational parameters of anaerobic digestion process such as sCOD, TVFA, DS, VS, and pH were analyzed throughout the incubation period. All experimental analysis for different measurements were conducted in triplicate.

### Analytical analysis

The performance of anaerobic digestion pretreated with low-frequency ultrasonic were assessed using the digestate samples withdrawn in predefined days within the incubation period. To this end, the digestate samples were centrifuged for 10 min at 3,000 r/min; the liquid portions were filtered from a filter pore size of 0.45 μm (PTFE-L) to eliminate any remaining solid particles. Next, the sCOD, pH, alkalinity, and total volatile fatty acids (TVFAs) were measured according to procedure described in follows. The soluble COD of different samples were measured by closed reflux method and Hach COD high range vials and DR 5000 Spectrophotometer. pH meter (WTW inoLab pH 720, Xylem—WTW, Germany) was used to measure the pH variation during the incubation period. The Nordmann method (DOC316.52.93087) method were used to determine the TVFA (as mg/L CH_3_COOH) and alkalinity (as mg/L CaCO_3_) by adjusting the pH by 0.1 N H_2_SO_4_ at endpoints of 5.0 and 4.4, respectively^27^. Furthermore, the total solids (TS) and volatile solids (VS) of the digestate samples collected from various anaerobic reactors were determined using the protocols specified in the standard methods for analyzing water and wastewater^28^.

### Anaerobic digestion energy assessment

According to Eqs. 1–3, the energy balance was approximated by estimating the energy used for mixing and heating and the energy produced from methane production:

Equations 1 and 2 depict the amount of electricity (in kJ/g VS removed) needed for mixing and increasing the temperature of the influent during digestion:

1 $${\text{E}}_{{{\text{i}}.{\text{electricity}}}} = \frac{{{\text{V}}*300*24}}{{VS\;removed}}$$

In this context, the variable Ei represents the amount of electricity required for mixing (measured in kJ/g VS removed), V stands for the working volume in cubic meters, and VS removed refers to the grams of volatile solids consumed during the incubation period^29,30^.

As for the energy input for raising the substrate temperature during the digestion process, Eq. 2 was employed:

2 $${\mathrm{E}}_{\mathrm{r}.\mathrm{electricity}}=\frac{\uprho *V*\gamma *24*0.8}{{VS\;removed}}$$

where, the variable Er represents the quantity of electricity required (measured in kJ/g VS removed), ρ stands for the density of the influent (in units of 1000 kg/m3), γ denotes the specific heat of the influent (4.18 kJ/kg °C), V refers to the working volume in cubic meters, and VS removed represents the grams of volatile solids consumed during the incubation period^29,30^.

Subsequently, Eq. 3 was utilized to calculate the energy output based on the methane produced during the incubation period:

3 $${\text{E}}_{0} = \frac{{{\text{P}}_{{CH_{4} }} *\varepsilon *\lambda _{m} }}{{VS\;removed}}$$

where E_0_ represents the energy output (measured in kJ/g VS removed), ${\mathrm{P}}_{{CH}_{4}}$ stands for the cumulative methane production during the incubation period (in units of m^3^), ε denotes the lower heating value of methane (35,800 kJ/m^3^CH^4^), λ_m represents the energy conversion factor of methane (0.9), and VS removed refers to the grams of volatile solids removed during the incubation period^31,32^.

## Results and discussion

The physico-chemical properties of raw LL used as the feedstock in anaerobic digestion were summarized in Table 1 summarizes. As per Table 1, the low biodegradability index (BOD_5_/COD = 0.25) and high pH (7.1) confirm the non-easily biodegradable of LL which require a pretreatment process before feeding to anaerobic digestion^2^. In addition, the low biodegradability index indicated that the LL employed in the current study is classified in middle-aged category^7^. In addition, the information on the physico-chemical characteristics of anaerobic digested sludge as the inoculum is given in Table 1. As seen in Table 1, the high VS/TS ratio (71%) of anaerobic digested sludge indicated the presence of desirable organic matter and microorganisms capable of degradation the high-chain TVFA in LL in order to facilitate the digestion process.

### Low-frequency ultrasonic process on RLL bioavailability

In the present study, the low-frequency ultrasonic pretreatment was performed in order to improve the bioavailability (sCOD and TVFA) of RLL before feeding to the anaerobic reactors. In addition, the roles of influencing parameters including ultrasonic density and ultrasonic time on RLL in pretreatment process were investigated. The ultrasonic and cavitation process improve the disintegration and consequently solubilization of suspended solids. In addition, the •OH oxidizing agent as the products of ultrasonic process lead to hydrolysis of long-chain TVFA to more bioavailable compounds which are more desirable for microorganisms in biological process^9,33^.

#### Influence of ultrasonic density on RLL bioavailability

**Figure 3:**
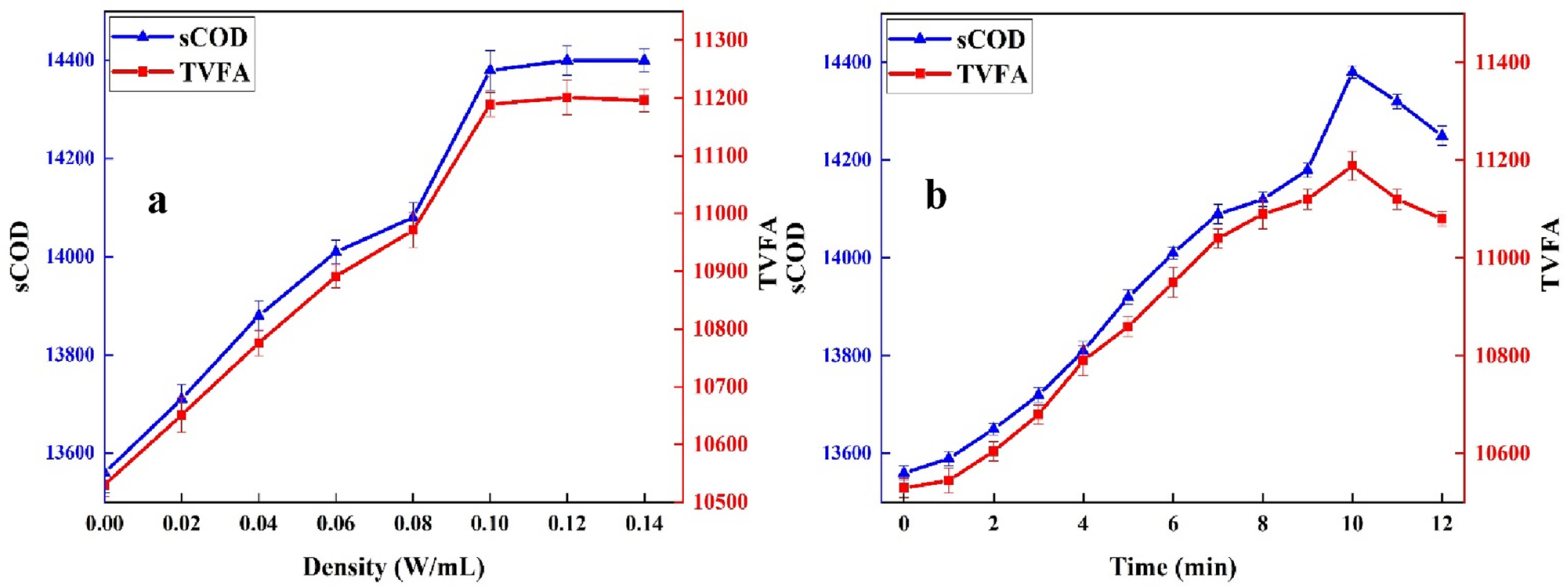
(**a**, **b**) This sentence describes the impact of ultrasonic density (**a**) and ultrasonic exposure time (**b**) on the biodegradability of landfill leachate during the ultrasonic pretreatment process.

The experimental study on enhanced RLL biodegradabaility (sCOD and TVFA) were conducted under the different ultrasonic density (UD) between 0.02 W/mL and 0.12 W/mL. The all experimental runs were conducted at the initial pH of RLL (7.1 ± 0.2). The changes of sCOD and TVFA as a function of ultrasonic density in low-frequency ultrasonic pretreatment process is presented in Fig. 3a,b indicate. According to Fig. 3a,b, a similar two-stage trend was observed for sCOD and TVFA variation with increased the ultrasonic density. As for sCOD variation, a increased values were observed when the ultrasonic density increased from 0.02 W/mL to 0.1 W/mL; the sCOD incresed from 13,560 mg/L to 14,380 mg/L. However, further increased the ultrasonic density led to constant increased value for sCOD; the sCOD increased only by 20 mg/L. Therefore, given the energy consumption and economical aspect of ultrasonic pretreatment process, the ultrasonic density equal to 0.1 W/mL was considered as optimum UD for further experiments. In case of TVFA, a similar trend was observed; the TVFA experienced a increasing trend from 10,530 to 11,189 mg/L when the UD increased from 0.02 to 0.1 W/mL. However, a constant variation was observed for further increased UD (see Fig. 3a).

The ultrasonic process due to hydrodynamic shear force and rapid collapse of microbulbbles in aquatic phase lead to disintegration of solids and organic matters found in landfill leachate. Furthermore, the disintegration of solids and organic matters accordingly improve the solubility of organic matters which indicate this change in increased sCOD^34,35^. As shown in Fig. 3a, ultrasonic irradiation can effieicntly solubolize the complex organic compounds and lead to higher bioavailability of RLL. In addition, the production of oxidizing radical such as •OH in the ultrasonic process can improve the biodegradability index of LL (TVFA) due to degradation of recalcitrant organic compounds^36^. A similar result was reported by Pretty Mori Budiman (2016), focusing improvement the photofermentative biohydrogen production using ultrasonic pretreatment of combined effluents from palm oil, pulp and paper mills. The authors reported an increased 16–252% sCOD when energy inputs increased from 48 to 1245 J/mL^35^.

#### The influence of ultrasonic time on RLL bioavailability

Figure 3b shows how the biodegradability of LL (as measured by sCOD and TVFA) is influenced by varying ultrasonic exposure times (ranging from 1 to 12 min), while holding the optimal ultrasonic density obtained in the previous step (UD = 0.1 W/mL) constant. As seen in Fig. 3a,b two-step trend was observed for sCOD and TVFA variation during the ultrasonic pretreatment; the sCOD experienced an increasing trend within the first 10 min and the highest sCOD was measured at 10 min (14,380 mg/L). However, with further increased the ultrasonic time to 12 min, the sCOD decreased by 130 mg/L. Therefore, ultrasonic time equal to 10 min was selected as the optimum ultrasonic time. In addition, a similar trend was observed for TVFA as the other biodegradability index of landfill leachate; the highest value of TVFA (11,189 mg/L) was observed at 10 min of ultrasonic exposure. It is believed that ultrasonic, depending on the solid content, improve the solubility and biodegradability of organic matters. However, after the optimal time, a decreasing trend is observed^9,35, 36^. Nilgun Ayman Oz (2014) reported a limited and negligible sCOD improvement after 2 min of ultrasonic irradiation from olıve mıll wastewater^36^.

In addition, Binay Kumar Tripathy (2019) reported that 15-min low-frequency ultrasonic irradiation (20 kHz) improved the BOD_5_/COD of mature LL from 0.033 to 0.142^9^. Overall, the UD and UT equal to 0.1 W/Ml and 10 min, respectively, were considered as the optimal operational parameters of ultrasonic pretreatment process for further experimental study and anaerobic digestion.

### Biogas upgrading process

**Figure 4:**
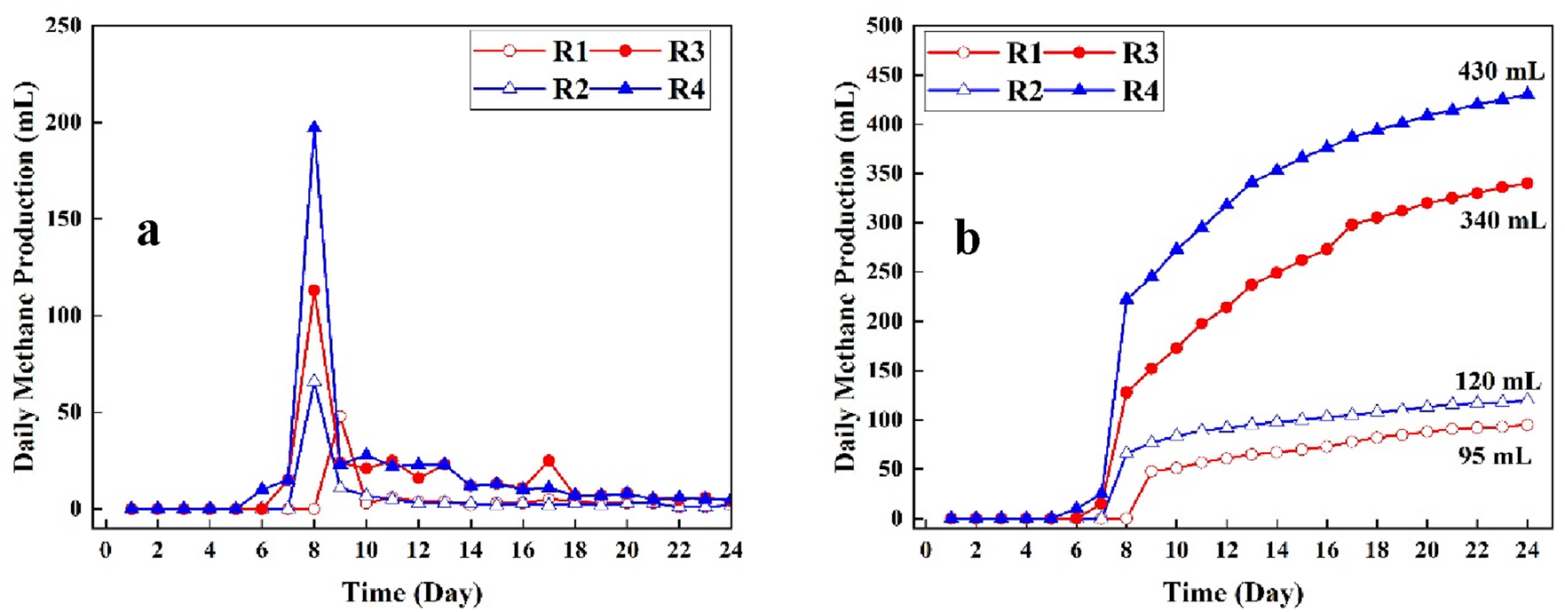
(**a**, **b**) The daily methane profile (**a**) and cumulative methane production (**b**).

The enhanced biogas production from LL pretreated by low-frequency ultrasonics for different volume ratios within 24 days of mesophilic anaerobic digestion were investigated in this stage. Figure 4a,b illustrates both the daily methane production and the cumulative methane production.

The daily profile of methane production and cumulative methane production for different volume ratios (10 and 15%) of LL pretreated with low-frequency ultrasonic and controls are shown in Fig. 4a,b. As seen in Fig. 4a, the initial and first production of methane for reactors pretreated with ultrasonic (R3 and R4) were observed at day 7 and 6, respectively. While, the lag phase for control reactors, the time required to observe the first methane production, were observed at day 9 and 8, respectively, for R1 and R2. These results obtained from the daily methane production indicated that low-frequency ultrasonic provides shorter lag phase, indicating the shorter incubation period and consequently lower cost for anaerobic digestion operational process. These results are in consistent with other studies focusing on enhanced methane production through LL pretreatment process both by ultrasonic and other pretreatment process^33,37^ (Li et al., 2018; Pasalari et al., 2021)). The highest daily methane production were observed after the lag phase. It ca be attributed to higher degradation of organic matters due to the hydrolysis stage of anaerobic digestion^38,39^. For instance, the highest daily methane production in R4 (197 mL) and R3 (113 mL) were observed at day 8. In addition, after the lag-phase period, a fluctuation was observed for different reactors feed with different ratio of LL pretreated with low-frequency ultrasonic and control within the incubation period (24 days). Figure 4b shows the cumulative methane production in anaerobic reactors fed with LL pretreated with low-frequency ultrasonic and controls. The cumulative methane production in R4 and R3, pretreated with low-frequency ultrasonic and 15 and 10% LL were estimated to be 430 and 340, respectively, which are 3.58 and 3.57 times more than the control (R2 and R1). Furthermore, the T90 (the time which is required to achieve the 90% of cumulative methane production in anaerobic digestion) in R3 and R4 were estimated to be day 22 and 21, respectively.

### Digestion performance

#### Soluble COD evolution

**Figure 5:**
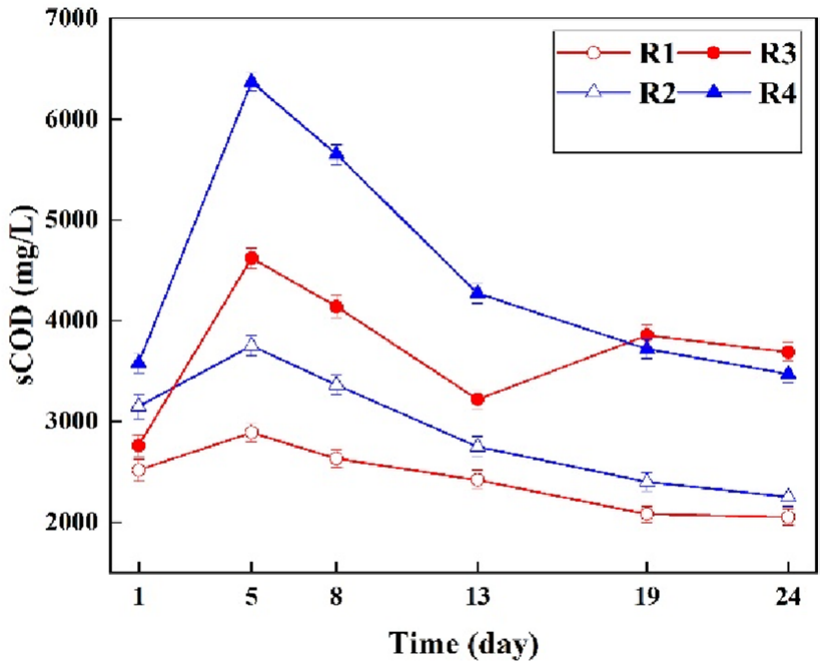
The sCOD variation of anaerobic reactors assisted with low-frequency ultrasonic.

The temporal changes of sCOD concentrations for different anaerobic reactors fed by LL pretreated with low-frequency ultrasonic are depicted in Fig. 5. According to Fig. 5, all different anaerobic reactors follows the same trend; the sCOD experienced an increasing trend in the first phase of anaerobic digestion (hydrolysis and acidogensis) and consequently a decreasing trend was observed due to conversion of organic matters to methane^36,39^.

According to Fig. 5, the higher values of sCOD concentration were observed in anaerobic reactor assisted with low-frequency ultrasonic pretreatment; the highest sCOD concentration (6370 mg/L) was found to be in R4, where highest volume of LL pretreated fed in. Therefore, the results indicated that low-frequency ultrasonic make desirable conditions for better solubilization of organic matter in raw LL and consequently anaerobic digestion process. For instance, the initial sCOD in R4 enhanced significantly from 3579 to 6370 mg/L within first 5 days, which is consistent with Pasalari et al. (2021), focused on improved methane production from LL pretreated with electrochemical oxidation process^2^. In addition, after completion the incubation period, the highest sCOD reduction in reactors pretreated with low-frequency ultrasonic were 900–2900 mg/L. While, these reduction values for controls were 840- 1500 mg/L.

#### pH and total volatile fatty acids (TVFA)

**Figure 6:**
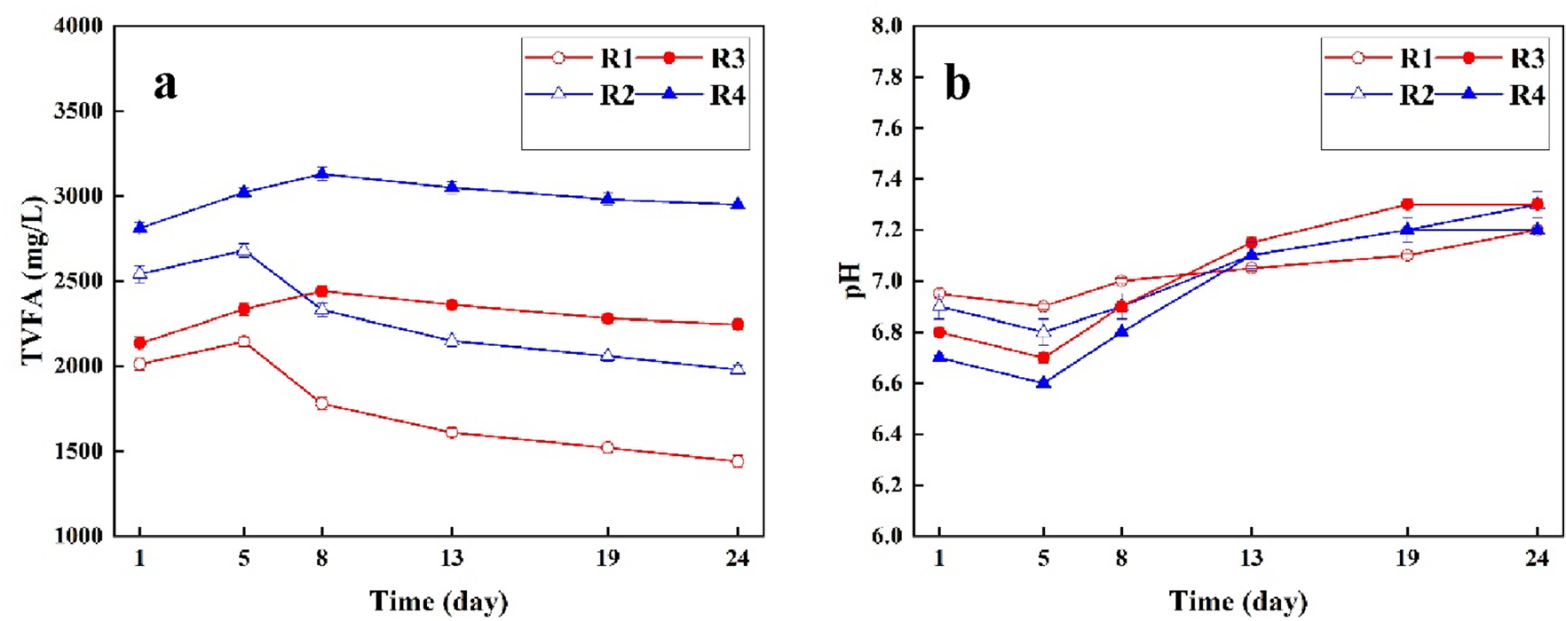
The TVFA (**a**) and pH (**b**) variation of anaerobic reactors assisted with low-frequency ultrasonic.

Figure 6a shows the changes of TVFA (measured as acetic acid) as the critical parameter in anaerobic digestion process for different reactors assisted with low-frequency ultrasonic and control. According to Fig. 6a, hydrolysis, acidogenesis, acetogenesis and methanogenesis as different phases of anaerobic digestion process are observed through TVFA variation. For instance, higher TVFA due to more organic matter degradation confirms the first stage of digestion process, hydrolysis step^1,40^. The highest TVFA was observed in R4 (3020 mg/L) followed by R3 (2234 mg/L). Generally, higher concentration of TVFA were observed in reactors pretreated with low-frequency ultrasonic compared to controls. Higher concentration of TVFA support the desirable mechanism of degradation assisted with ultrasonic process^41^. After the lag phase and increasing trend, TVFA experienced a decreasing trend due to methane production which are produced by methanogenis activity in anaerobic process^42^.

Figure 6b shows the temporal pH variation in different anaerobic reactors pretreated with low-frequency ultrasonic and controls. As observed in Fig. 6a,b same trend was observed for different anaerobic reactors; the pH experienced a decreasing trend due to higher TVFA as function of hydrolysis in the first stage of anaerobic digestion. It is worth mentioning that a greater reduction in pH was observed in R4 when a larger volume ratio of pretreated LL was fed in. Nonetheless, throughout the rest of the incubation period, the pH levels in the various anaerobic reactors remained within the desired range of 7.2–7.4. The results are in consistent Pasalari et al., 2021; Montusiewicz et al., 2018, Ghanem et al., 2001, focused on the digestion of landfill leachate (Montusiewicz et al., 2018)^2^[67].

### Energy balance in anaerobic digestion process

Energy recovery and energy consumption in different anaerobic reactors pretreated with low-frequency ultrasonic were evaluated in order to evaluate the energy balance. Table 2 provides a summary of the energy evaluation conducted on the anaerobic reactors with ultrasonic assistance, as well as the control group.

**Table 2:** Energy balance in different anaerobic reactors pretreated and controls.

	R1	R2	R3	R4
Energy consumption (Mixing) (kJ/g VS removed)	0.65	0.58	0.46	0.41
Energy consumption (Temperature) (kJ/g VS removed)	4.96	4.96	4.96	4.96
Output energy (kJ/ g VS removed)	2.78	3.09	6.99	7.98
Total	− 2.83	− 2.43	+ 1.57	+ 2.61

According to Table 2, the output energy for different anaerobic reactors assisted with low-frequency ultrasonic were between + 6.99 and + 7.98 kJ/g VS removed; those for controls were 2.78–3.09 kJ/g VS removed. The results proves that low-frequency ultrasonic leads to more production of biogas and consequently the overcome of energy production over the energy required to operate the anaerobic digestion during the incubation period. The results presented here are comparable with Pasalari et al. (2021)^2^ focused on biogas improvement from the LL by electrochemical oxidation in anaerobic co-digestion (3.60–25.55 kJ/g VS_removed_) and Benyi Xiao (2018) with special focus on single and two-stage thermophilic digestion of food waste (16.67–17.28 kJ/g VS_removed_). The lower energy production in this study compared to mentioned studies are the using of codigestion process and supplementary substrates in co-digestion process and take advantages of food waste with higher volatile solids.

## Conclusion

The present study indicated that low-frequency ultrasonic is a feasible pretreatment to improve the biogas production from LL in anaerobic digestion and energy recovery. The low-frequency ultrasonic can incorporate to achieve higher bioavailability of LL and shorten the lag-phase period, which consequently lower the operation cost. The cumulative methane production in the anaerobic reactors that received assistance from low-frequency ultrasonic treatment was 3.57–3.58 times higher than that of the control group. Additionally, the energy evaluation demonstrated that the anaerobic digestion reactors that were assisted with low-frequency ultrasonic pretreatment exhibited an increase in output energy and energy recovery within the range of + 6.99 to + 7.98 kJ/g VS removed. However, more research is required to comprehensively understand the methanogenic pathways occurred by predominance microorganisms.

## Data Availability

The datasets generated and analyzed during the current study available from the corresponding author on reasonable request.
